# Predictive Gene Signatures: Molecular Markers Distinguishing Colon Adenomatous Polyp and Carcinoma

**DOI:** 10.1371/journal.pone.0113071

**Published:** 2014-11-25

**Authors:** Janice E. Drew, Andrew J. Farquharson, Claus Dieter Mayer, Hollie F. Vase, Philip J. Coates, Robert J. Steele, Francis A. Carey

**Affiliations:** 1 Metabolic Health, Rowett Institute of Nutrition and Health, University of Aberdeen, Aberdeen, AB21 9SB, Scotland; 2 Biomathematics and Statistics Scotland, University of Aberdeen, Aberdeen, AB21 9SB, Scotland; 3 Ninewells Hospital and Medical School, Dundee, DD1 9SU, Scotland; Howard University, United States of America

## Abstract

Cancers exhibit abnormal molecular signatures associated with disease initiation and progression. Molecular signatures could improve cancer screening, detection, drug development and selection of appropriate drug therapies for individual patients. Typically only very small amounts of tissue are available from patients for analysis and biopsy samples exhibit broad heterogeneity that cannot be captured using a single marker. This report details application of an in-house custom designed GenomeLab System multiplex gene expression assay, the hCellMarkerPlex, to assess predictive gene signatures of normal, adenomatous polyp and carcinoma colon tissue using archived tissue bank material. The hCellMarkerPlex incorporates twenty-one gene markers: epithelial (EZR, KRT18, NOX1, SLC9A2), proliferation (PCNA, CCND1, MS4A12), differentiation (B4GANLT2, CDX1, CDX2), apoptotic (CASP3, NOX1, NTN1), fibroblast (FSP1, COL1A1), structural (ACTG2, CNN1, DES), gene transcription (HDAC1), stem cell (LGR5), endothelial (VWF) and mucin production (MUC2). Gene signatures distinguished normal, adenomatous polyp and carcinoma. Individual gene targets significantly contributing to molecular tissue types, classifier genes, were further characterised using real-time PCR, *in-situ* hybridisation and immunohistochemistry revealing aberrant epithelial expression of MS4A12, LGR5 CDX2, NOX1 and SLC9A2 prior to development of carcinoma. Identified gene signatures identify aberrant epithelial expression of genes prior to cancer development using in-house custom designed gene expression multiplex assays. This approach may be used to assist in objective classification of disease initiation, staging, progression and therapeutic responses using biopsy material.

## Introduction

Colorectal cancer is the fourth most common cause of death from cancer, accounting for 8% of all cancer deaths [1]. The majority of colorectal cancers arise from adenomatous polyps. With the advent of population screening large numbers of asymptomatic individuals are being shown to have adenomas [2]. Clinical follow up of these individuals is a major challenge for health services. Polyp size and number are the only reliable predictors for screened patients at risk of future neoplastic disease, but even these are not a sensitive indicator and large numbers of patients who will never develop a cancer are currently followed up by colonoscopy. Dysplasia, a morphological assessment of cytological and architectural variation from normal is a better marker of progression [3], but is prone to inter-observer variation and objective biomarkers are needed. This would greatly assist in objective stratification of those at risk of progression to malignancy, permitting a more targeted approach to surveillance of the increasing number of individuals identified with colon polyps.

Disruption of cellular homeostasis is a fundamental feature of the events that lead to carcinogenesis [4], [5]. Evidence has demonstrated that carcinogenesis proceeds in intermediate stages reflecting accumulation of mutations that drive altered cellular behaviours, transforming normal cells to malignant derivatives [4], [5]. This is characterised by altered gene transcription controlling aspects of cell homeostasis associated with cell proliferation, differentiation and apoptosis [4], [5]. Adenomatous polyps are recognised as a potential precursor of malignant transformation and exhibit increased proliferation of stem cells located at the base of colon crypts. These progenitor cells generate the epithelium that lines the colon, which becomes distorted as a consequence of hyper-proliferation, with concomitant reduced differentiation and apoptosis [4], [5]. Altered tissue micro-architecture also becomes apparent with alteration of cellular structural components and the associated microenvironment containing inflammatory cells [6], fibroblasts [7] and endothelial [8] cells. Cellular processes of differentiated cells are disrupted with altered function of endocrine cells within the epithelium [9] and increased angiogenesis [8].

Capturing the profound changes in transcriptional regulation that occur in adenomatous polyps and cancer presents a potential means of objectively assessing pre-malignant changes in tissues that predispose adenomatous polyps to malignant transformation. This has prompted interrogation of the abnormal gene expression associated with initiation and progression of colorectal cancer using high throughput gene expression screening technologies, such as microarray, revealing complex altered profiles of gene expression [10], [11]. It is apparent that human colon pathology samples exhibit a broad diversity that cannot be captured using single biomarkers. It is necessary to distil the discovery of these broad molecular signatures into smaller gene sets of appropriate density to generate predictive gene signature assays that have clinical utility to permit a comprehensive insight on dysplasia and decipher information relating to cancer initiation, staging and progression. Further problems are encountered with typically only very small amounts of tissue of variable quality being available for analysis from patient biopsy samples.

These challenges are being addressed in our lab using strategies to design in-house bespoke assays incorporating multiple gene markers to conduct gene expression profiling using the GenomeLab System technology platform [12]. Designed multiplex assays can incorporate up to thirty gene targets and can be conducted using very small tissue samples to generate gene signature profiles from biopsy tissue [12]. Previous studies have demonstrated that the persistent technical difficulties presented by multiplexed quantitative real-time PCR [13], [14] can be overcome using the GenomeLab System [12].

This report describes the development and evaluation of an in–house custom designed cell marker multiplex, the hCellMarkerPlex, incorporating twenty-one gene markers of key cellular processes and aspects of cell maintenance altered in colon carcinogenesis (GeneCards http://www.genecards.org/). Archived colon biopsy tissues collected from patients undergoing routine bowel screening were assayed using the hCellMarkerPlex to determine distinguishing gene signature profiles identifying normal, adenomatous polyp and carcinoma gene signatures. The aim was to identify potential classifier genes contributing to different tissue pathology status that can be used to apply custom designed assays that can be applied to assist in objective prospective classification of colon pathology samples.

## Materials and Methods

### 2.1 Biopsy specimens

Colon tissue samples (normal, adenomatous polyp and carcinoma) were obtained from the Tayside Tissue Bank, Dundee, Scotland. The archived tissues were obtained from patients attending for colonoscopy or surgery at Ninewells Hospital, Dundee. All patients consented for research use of tissues using the forms approved by Tayside Local Research Ethics Committee through the Tayside Tissue Bank. Tissue samples were frozen and stored at −80°C prior to analysis by GeXP assay and *in situ* hybridisation. Formalin fixed diagnostic paraffin-embedded tissue blocks were also stored and available for immunohistochemistry (IHC). All tissue samples were diagnosed and graded for dysplasia using conventional criteria within the pathology department at Ninewells Hospital, Dundee (Table S1).

### 2.2 Total RNA extraction

RNA was extracted from approximately 10 mg of each colon specimen using an RNeasy Mini Kit (Qiagen, Crawley, UK), incorporating a DNase digestion. All of the extracted RNA samples were analysed using the Agilent Bioanalyser (Agilent Technologies, Bracknell, UK) to obtain RIN values allowing assessment of total RNA quality. Quantitation for downstream processing was assessed using a Nanodrop spectrophotometer (Nanodrop Technologies). Total RNA was aliquoted and stored at −80°C prior to analysis of gene expression.

### 2.3 Selection of cell marker gene targets to be incorporated in the custom designed multiplex GeXP assay, the hCellMarkerPlex

Twenty-one gene markers expressed in different colon cell types and associated with specific cellular processes in the colon that are disrupted in response to pathology were selected using GeneCards (http://www.genecards.org/) and were incorporated into an in-house custom designed GeXP assay, the hCellMarkerPlex. The hCellMarkerPlex represents 6 cell marker groups; epithelial (*EZR*, *KRT18*, *SLC9A2*), proliferation (*PCNA*, *CCND1*, *MS4A12*), differentiation (*B4GANLT2*, *CDX1*, *CDX2*), apoptotic (*CASP3*, *NOX1* and *NTN1*), fibroblast (*FSP1* and *COL1A1*) and structural (*ACTG2*, *CNN1* and *DES*) together with gene transcription (*HDAC1*), stem cell (*LGR5*), endothelial (*VWF*) and mucin production (*MUC2*) markers (Table S2) (see GeneCards http://www.genecards.org/for further information on selected genes). The gene target accession numbers were obtained from the NCBI website (http://www.ncbi.nlm.nih.gov/nuccore) and were loaded into the Genome Lab GeXP database, together with reference genes (*UBE2D2* and *B2M*) and a synthetic reference control transcript (*Kan(r)* supplied with the GeXP assay kit (Beckman Coulter, UK) (Table S2). *B2M* has been validated as a reference marker for colon tissue in previous studies [12]. Stable expression of *UBE2D2* was observed in previous gene expression analysis of human colon tissues using the Beckman Coulter human Reference Plex (data not published). The third reference gene is an external synthetic reference control transcript Kan (supplied with the GeXP assay kit, Beckman Coulter, UK) used to spike each reaction. Two reference genes were selected for normalisation as recommended for relative quantitative gene expression analysis [15], [16].

### 2.4 GeXP hCellMarkerplex primer assay design

The GenomeLab eXpress designer GeXP Software (Beckman Coulter, UK) was used to identify suitable gene specific primers for reverse transcription and PCR amplification (Table S2) as previously described [12]. Reverse PCR primers were designed with a 3′ gene specific sequence and a 5′end consisting of 19 bases of universal priming sequence. The forward PCR primers were designed with a 3′ gene specific sequence and a 5′end consisting of a different 18-nucleotide universal priming sequence. The gene specific primers were designed to generate PCR amplicons that differ in size by 4–7 base pairs, ranging in size from 137–325 (Table S2). Primer sequences were evaluated using BLAST searches to ensure specific amplification of the designed PCR fragments. User-defined regions of the listed sequences were selected for primer design where targets were known to be members of a gene family to exclude homologous regions likely to cause mis-priming and aberrant amplification. Primers with universal sequences were purchased from Sigma-Genosys (UK).

### 2.5 Optimisation of the GeXP hCellMarkerPlex

The hCellMarkerPlex was optimised using total RNA extracted from normal colon tissue. Total RNA (50 ng) was reverse transcribed using the hCellMarkerPlex reverse primer mix and the Genome Lab GeXP start Kit (Beckman Coulter) according to the manufacturer's instructions. Single gene specific reverse primers were initially diluted with nuclease-free water to a concentration of 500 nM in each singleplex assay with each of the forward primers (500 nM) to establish amplification of a single peak of the expected size. A multiplex mix of forward primers was then applied, followed by attenuation of reverse primer concentrations. Attenuation of signals beyond the linear range was achieved by altering reverse primer concentration (in the range 15.6 nM to 1000 nM) according to manufacturer instructions. Primer concentrations used in optimised multiplex are listed (Table S2). Reverse transcription and PCR reactions were performed in a thermal-cycler (G Storm GS-I, GRI Ltd, UK) as described previously [12]. No template and no reverse transcriptase controls were conducted to ensure the absence of non-specific reaction products. GeXP PCR reactions products were then processed as described previously [12] for capillary electrophoresis and fragment separation using a CEQ 8800 (Beckman) as described previously [12].

Following CEQ analysis the raw data was analysed using the Fragment Analysis module of the GenomeLab System software (Beckman). A size exclusion filter appropriate for the custom designed hCellMarkerPlex was applied to determine expected size fragments. The fragment data, peak height and peak areas were then imported to the eXpress Analysis module of the eXpress Profiler software (Beckman) and analysed as described previously [12]. Analysis of the signal intensity data was conducted using geNorm (http://medgen.ugent.be/genorm/) to establish the most stably expressed transcript for normalisation purposes.

### 2.6 hCellMarkerPlex quantitative gene expression profiling of colon biopsy tissues

The hCellMarkerPlex was then applied to total RNA (50 ng in triplicate) extracted from human colon normal (n = 30), adenoma (n = 20) and carcinoma (n = 24) tissues, consisting of matched normal, adenoma and carcinoma (n = 14), matched normal and adenoma (n = 6) and matched normal and carcinoma (n = 10) (Table S1). Reactions were conducted and analysed as described above. Raw data were exported using the GenomeLab express analysis bygene export option and normalised to each of the reference genes, *UBE2D2* and *B2M*, incorporated in the multiplex. The quantitative gene expression profiles generated by hCellMarkerPlex assay were measured.

### 2.7 SYBR real-time PCR Assay

Complementary cDNA templates for real-time PCR assays were prepared from Superscript II (Invitrogen) reverse transcribed total RNA (0.5 µg). SYBR real-time PCR analysis was performed, according to the manufacturer's instructions, using Superarray Bioscience Corporation SYBR green master mix (Tebu-Bio, UK). Two human *MS4A12* primer pairs (Sigma-Genosys, UK) were designed to amplify either *MS4A12* variant 1 (5′-gcaaaggcactaggggtgatcca-3′ and 5′-ggccaccccagaatgggtatcca-3′) or *MS4A12* variant 2 (5′-ggcactagggtttattatctctggc-3′ and 5′-tcccaggctgcctttcaccag-3′), together with a primer pair for the selected reference gene *UBE2D2* 5′-cagcacagtgttcagcaggt-3′ and 5′-tgaaggggtaatctgttggg-3′.

All real-time PCR assays were performed using the ABI-7500Fast (Applied Biosystems, UK). A two step cycling programme with an initial step of 10 minutes at 95°C to activate the Hotstart DNA polymerase being used, followed by 40 cycles of 95°C for 15 sec, 55°C for 30 sec and 72°C 30 sec was used for *MS4A12* variant 1 and *UBE2D2*, with *MS4A12* variant two being annealed at 53°C. The threshold cycle number (*C*_t_) was measured using the ABI7500Fast associated software (Applied Biosystems). Transcript levels relative to the reference gene, *UBE2D2*, were calculated (Δ*C*_t_). Fold expression changes between experimental groups relative to *UBE2D2* were calculated from the ΔΔ*C*_t_ values.

### 2.8 *In situ* hybridisation

Frozen sections (10 µm) were cut from normal, adenomatous polyps and carcinoma tissue (n = 5). *In situ* hybridisation was performed as described previously [17]. The riboprobe templates for *in-situ* localisation were generated by PCR using the following primer pairs: MS4A12 5′-tctggtgaaaggcagcctggga-3′ and 5′-acagccatcattagcgaccaacc-3′, SLC9A2 5′-ctccccctgcaatgaagactgat-3′and 5′-agcaccccaccgattcccacaac-3′, NOX1 5′- ctgtgcccgagcgtctgc-3′ and 5′- caatgccgtgaatccctaagc-3′, CDX2 5′- tggccggcagcgtatgg-3′ and 5′- tccggatggtgatgtagcgactgt-3′ and LGR5 5′- aatccccgtccaggcttttag-3′ and 5′- gaggcaccattcagagtcagt-3′. The primers generated the PCR products of 484 bp MS4A12, 518 bp NOX1, 454 bp CDX2, 464 bp SLC9A2 and 420 bp LGR5 that were cloned into pGEM-T easy (Promega, UK) or pBluescript (Stratagene, UK) and riboprobe template sequences were verified using a Beckman CEQ8000 Genetic Analyser. Antisense and sense probes were synthesised from the linearised template by *in vitro* transcription using RNA T7 and T3 polymerases as appropriate in the presence of ^35^S-alpha-thio-UTP (NEN; 1000 Ci/mmol). Size and quality of the ^35^S-alpha-thio-UTP riboprobes were assessed by formaldehyde gel electrophoresis and northern blotting prior to use. Tissue sections were hybridised with radiolabelled riboprobes at 58°C and washed to 0.1×SSC at 60°C. Hybridised sections were assessed initially using a Fuji phosphorimager and AIDA Image Analyser software (Raytest Isotopenmebgerate GmBH, Germany) prior to coating with LM-1 liquid emulsion (Amersham Pharmacia Biotech Ltd., UK) and staining with toluidine blue. The sense riboprobe hybridised sections were examined to assess any non-specific hybridisation.

### 2.9 Immunohistochemistry

Paraffin embedded tissue sections (4 µm) of normal, adenomatous polyp and carcinoma (n = 8) were de-paraffinised in Histoclear (National Diagnostics) and rehydrated through a graded alcohol series. Microwave-based antigen retrieval was conducted using10 mM citric acid buffer (pH 6.0). Sections were microwaved in a pressure cooker for 15 min prior to immunostaining on a DAKO autostainer using Vectastain ABC kits (Vector Labs) according to the manufacturer's protocol. Sections were blocked in either normal goat or horse serum containing 10% (v/v) stock avidin solution (Vector Labs) for 20 minutes followed by a 1 hour incubation with CDX2 antibody (Leica Microsystems) including 10% (v/v) from stock biotin solution (Vector Labs) to reduce non-specific background staining. Sections were incubated with either biotinylated anti-mouse (for monoclonal antibodies) antibody for 30 minutes followed by Vectastain® Elite ABC reagent for another 30 min. Liquid Diaminobenzidine (DAB) (DAKO) was used as a chromogenic agent for 5 minutes and sections were counterstained with Mayer's haematoxylin. Negative controls were processed without addition of the primary antibody. Positive staining was assessed morphologically by expected cellular compartment stained (e.g. nucleus for CDX2). Intensity of staining was scored on a semi-quantitative scale designated as follows: + − detectable nuclear staining, weak, ++ − easily visible nuclear staining, +++ − strong staining and n/a – no adenoma tissue in histological section.

### 2.10 Statistical analysis

Principal Component Analysis (PCA) was performed using SIMCA-P+12.0 software (MKS Instruments UK Ltd, Cheshire) on normalised and scaled data from hCellMarkerPlex assay of the patient tissue donors to assess expression patterns associated with normal, adenomatous polyp or carcinoma tissues. The same software was used to perform an Orthogonal Partial Least Squares Discriminant Analysis (OPLS-DA) [18], [19]. Similar to PCA Partial Least Squares tries to find linear combinations of variables, but conversely maximises covariance, rather than variance, with a response variable. In PLS discriminant analysis this response is of categorical nature (in our case the sample classes normal, adenomatous polyp and carcinoma) and the components obtained are chosen such that they can discriminate between the different categories. OPLS-DA is a variation of this method in which the matrix of explanatory variables (here the gene expression matrix) is first decomposed into a part that is orthogonal (unpredictive) to the response and another that is predictive. This approach improves the interpretability of the results and is widely used in metabolomics studies [18]. Jiang *et al*. [20] use this technique in a similar context and detail the OPLS-DA approach.

The Results from a linear discriminant analysis were used to assess quality of hCellMarkerPlex gene expression profiling data from RNA templates with different RIN values Gene expression levels were compared using a linear mixed model in Genstat v13.2 (VSN International Ltd., Hemel Hempstead, UK) (significance level 0.05). The analysis was conducted on a log scale if Genstat output data identified skewed effects for a variable.

## Results

### 3.1 Design and optimisation of the hCellMarkerPlex

Each gene specific primer pair was initially tested in a single-plex reaction. This determined that a single peak of the expected size was generated, with no spurious fragments produced. A multiplex primer mix of selected gene specific primers, the hCellMarkerPlex was then prepared for multiplex gene expression analysis. Attenuation was performed and primer concentrations giving optimal signal detection for application of the hCellMarkerPlex to the colon biopsy tissues were determined as listed in Table S2.

### 3.2 Patient biopsy specimens

The average age of patients was 65 years (range 37–82) (Table S1). Eleven patients were female and nineteen were male. Cancers (Dukes A, B, C or C1) originated on proximal, right side (ileum, cecum and ascending colon), hepatic flexure, transverse and distal, left side (descending colon, sigmoid and rectum) of the gut (Table S1). Adenomatous polyps originated from ascending colon, hepatic flexure, sigmoid and rectum and were classified as adenoma, tubulovillous adenoma, tubular adenoma and sessile serrated polyp (Table S1).

### 3.3 Gene expression profiling of cell marker genes in colon biopsy specimens

Total RNA RIN values ranged from 2–10, indicating variations in quality (Table S1). However, it proved possible to obtain hCellMarkerPlex gene expression profiles from all RNA samples. Analysis of the hCellMarkerPlex gene expression data using geNORM [21] determined that *UBE2D2* exhibited the most stable expression across the tissue samples. Subsequently all gene expression data was normalised using *UBE2D2* as a reference gene.

Linear discriminant analysis conducted on normalised hCellMarkerPlex gene expression data provided assessment of an appropriate cut off RIN value to exclude gene expression profiles that were adversely affected by RNA quality. Linear discriminant analysis identified that hCellMarkerPlex assay of RNA samples with RIN≥5 did not significantly differ in discriminating tissue pathology. Consequently, hCellMarkerPlex data derived from RNA samples with RIN≥5 were selected for further analysis.

Principal component analysis (PCA) was applied to the normalised gene expression data obtained from total RNA samples of RIN≥5 revealing that normal, adenomatous polyp and carcinoma tissues were associated with characteristic gene expression profiles obtained by hCellMarkerPlex assay. Two apparent anomalous carcinoma tissue expression profiles were identified clustering with normal biopsy expression profiles in this initial principal component analysis. The appropriate patient samples were retrieved from the Tayside Tissue Bank and subjected to additional pathological analysis. This resulted in reassigning one of the patient tissue samples as normal. The second carcinoma sample was observed to exhibit highly variant degrees of dysplasia. Consequently, the material used for RNA extraction from this particular biopsy may have largely consisted of tissue with a high degree of similarity to the normal gene expression profiles obtained by hCellMarkerPlex assay. These two anomalous samples were subsequently removed from the data set prior to further analysis.

**Figure 1:**
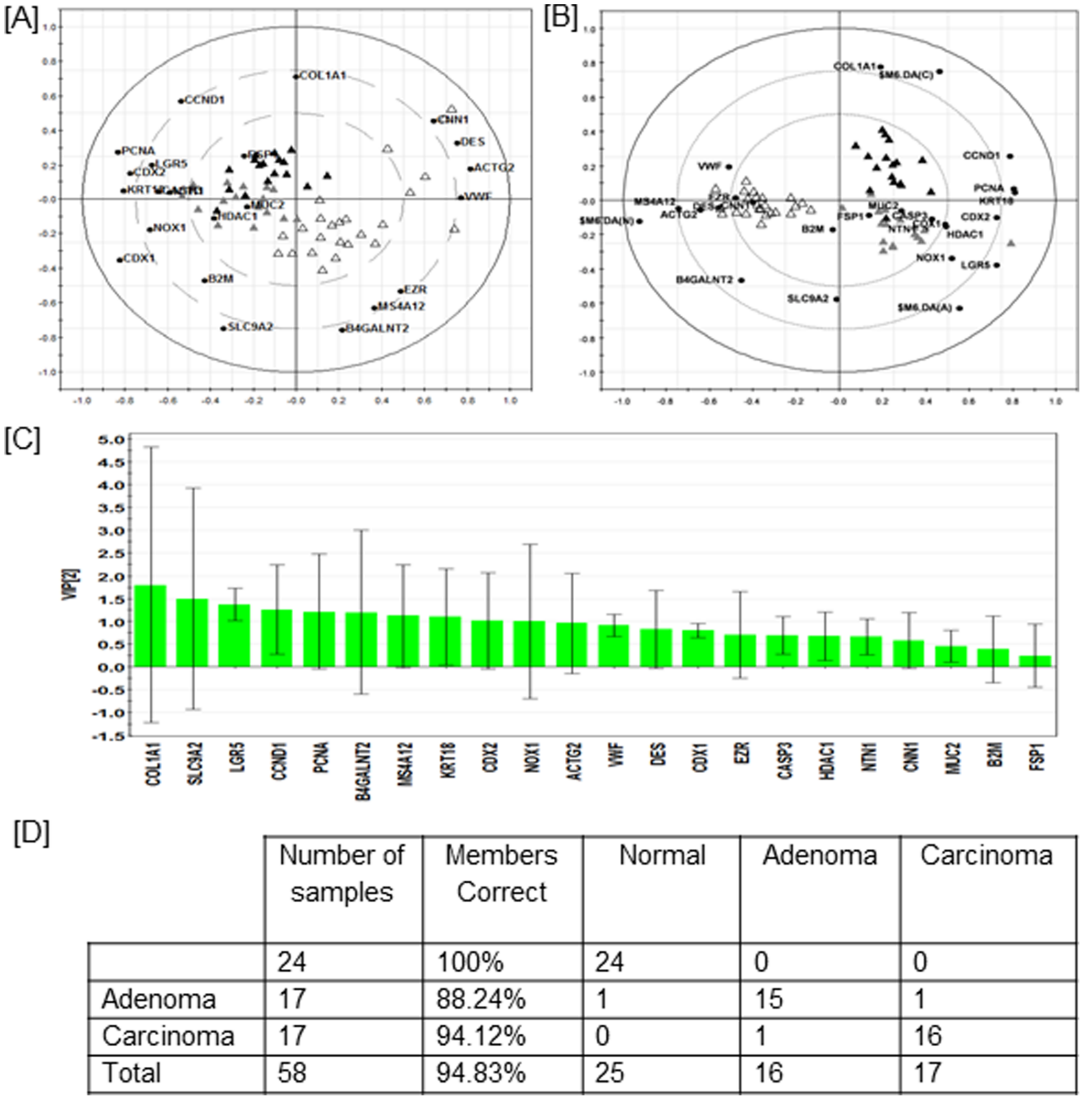
Multivariate discriminant analysis of the *UBE2D2* normalised gene GeXP hCellMarkerPlex data from human colon normal (white triangle) (n = 24), adenomatous polyp (grey triangle) (n = 17) and carcinoma (black triangle) (n = 19) tissues. Information on the gene symbols on the biplot is available in Table S2. (A) Principal component analysis (PCA) biplot permits visualisation of inherent clustering patterns of individual tissue samples and associated gene expression levels. (B) Orthogonal Partial Least Squares Discriminant Analysis (OPLS-DA) was applied to fit a 2-class supervised model maximising covariance and discriminating gene expression profiles associated with the different tissues sample types; the biplot shows scores and loadings as well as the regression coefficients best explaining each class ($M4.DA(N),$M4.DA(A),$M4.DA(C). (C) Rank of importance of cell marker genes within the OPLS-DA. (D) Matrix showing the associated misclassification rates.

A biplot of the PCA was constructed to further inform on associations between gene expression profiles and biopsy type (Figure 1A). The biplot reveals specific genes within the hCellMarkerPlex that have similar pattern of up/down regulation in each of the different biopsy samples, normal, adenomatous polyp or carcinoma, indicating potential classifier genes that contribute to classification of the different pathological tissue types used in the study.

Normal tissue is characterised by higher expression levels of *ACTG2*, *VWF*, *EZR*, *B4GALNT2*, *CNN1*, *DES* and *MS4A12* and lower levels of *NOX1*, *HDAC1*, *CCND1*, *LGR5*, *PCNA*, *CDX1*, *KRT18*, *NTN1*, *CDX2* and *CASP3* when compared to adenomatous polyp or carcinoma tissues (Figure 1). Carcinoma tissue is distinguished by significantly lower levels of *SLC9A2* and increased *COL1AI* compared to either normal or adenomatous polyp tissues (Figure 1A).

**Figure 2:**
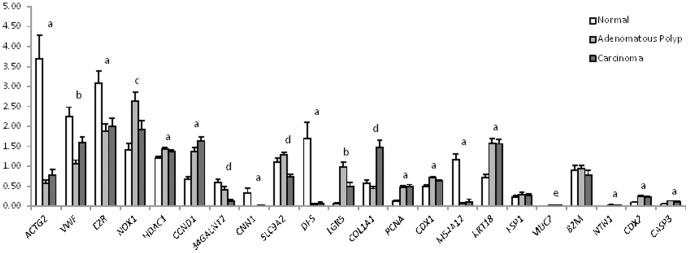
Relative gene expression levels in human colon normal, adenomatous polyp and carcinoma tissue generated using the GeXP hCellMarkerPlex assay. Gene expression is normalised to internal reference gene *UBE2D2* in the hCellMarkerPlex. The letters indicate significant (p<0.05) difference in gene expression between ‘a’ normal (n = 24) and either adenomatous polyp (n = 17) or carcinoma (n = 19), ‘b’ normal, adenomatous polyp and carcinoma, ‘c’ normal and carcinoma, ‘d’ normal and adenomatous polyp and ‘e’ carcinoma and either normal of adenomatous polyp.

Statistical analysis using a mixed linear model established genes showing significantly altered patterns of mean expression levels associated with pathology. *ACTG2*, *EZR*, *CNN1*, *DES*, *MS4A12* and *NTN1* are all expressed at significantly higher levels in normal tissue compared to adenomatous polyp or carcinoma tissues (Figure 2). Conversely, *HDAC1*, *CCDN1*, *PCNA*, *CDX1*, *KRT18*, *CDX2* and *CASP3* are all expressed at significantly lower levels in normal tissue compared to adenomatous polyp or carcinoma tissues (Figure 2). *VWF*, and *LGR5* had significant differences in mean expression levels in all three biopsy tissues (Figure 2). *NOX1* expression was significantly higher in adenomatous polyp tissue compared to either normal or carcinoma (Figure 2). *B4GALNT2*, *SLC9A2* and *COL1A1* were significantly altered in carcinoma compared to normal or adenomatous polyp tissue. *MUC2* expression was significantly lower in adenomatous polyps compared to normal, but not carcinoma (Figure 1A). *FSP1* and *B2M* did not show significantly altered expression patterns associated with tissue pathology (Figure 2).

Both the PCA (Figure 1A) and the linear mixed model (Figure 2) results show that tissue type is a major source of variation for the gene expression data of hCellMarkerPlex assay, but these analyses do not tell us whether it is possible to classify a sample as normal, adenoma or carcinoma based on the expression data only. To answer this question OPLS-DA was used. Figure 1B shows the corresponding bi-plot, in which component one separates the normal samples from the rest, whereas component two distinguishes between adenoma and carcinoma samples. The bi-plot together with the variable importance plot (Figure 1C) shows that it is mainly the difference between *COL1A1* and *SLC9A* that discriminates between adenoma and carcinoma, whereas high values of *CCND1, PGNA, KRTA6* and *LGR5* are indicative of abnormal tissues. The associated matrix (Figure 1D) shows overall 55 out of the 58 samples were correctly classified by this method demonstrating the potential of the hCellMarkerPlex assay to be developed into a diagnostic tool.

### 3.4 Expression of long and short MS4A12 variants

**Figure 3:**
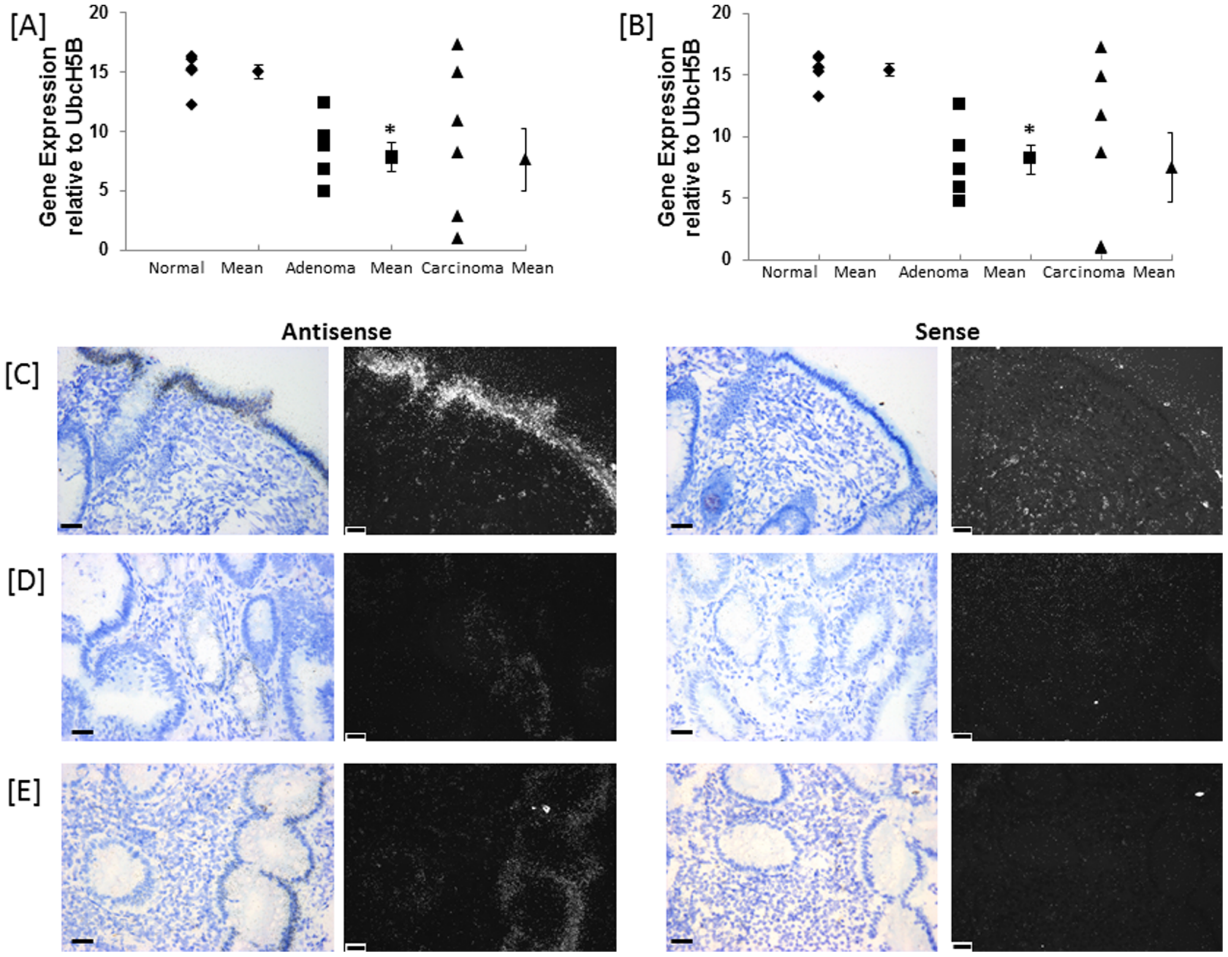
Gene expression of (A) long and (B) short form variants of *MS4A12* in human colon normal, adenomatous polyp and carcinoma tissue. Gene expression is normalised to reference gene *UBE2D2*. The asterisk (*) indicates significant decrease in expression levels of adenomatous polyp compared to normal, p<0.005. (C) – (D). *In situ* hybridisation of *MS4A12* transcripts in human colon (C) normal, (D) adenomatous polyp and (E) carcinoma. Emulsion autoradiographs showing expression of *MS4A12* at luminal epithelial surface (ep) of normal (C) in bright field and corresponding dark field images in antisense (left) and sense (right) hybridised tissue sections (n = 5). *MS4A12* is largely absent in adenomatous polyp (D) and localised in discrete areas of epithelium in carcinoma (E). Bar = 20 µm.

The hCellMarkerPlex assay does not discriminate between the long (NM_017716.2) and short (NM_00164470.1) variants of *MS4A12*. Further validation of *MS4A12* gene expression was conducted using SYBR real-time PCR with primer assays specific for the long (NM_017716.2) and short (NM_00164470.1) *MS4A12* variants in a subset (n = 6) of matched normal, adenoma and carcinoma colon patient biopsy samples. The hCellMarkerPlex expression pattern of significant reduced expression in adenoma compared to normal tissue and variable expression in carcinoma was validated by the SYBR real-time PCR analysis. Both long (Figure 3A) and short (Figure 3B) *MS4A12* variants show similar altered patterns of expression associated with pathology.

### 3.5 Localisation of selected classifier gene target expression in normal colon, adenomatous polyp and carcinoma

**Figure 4:**
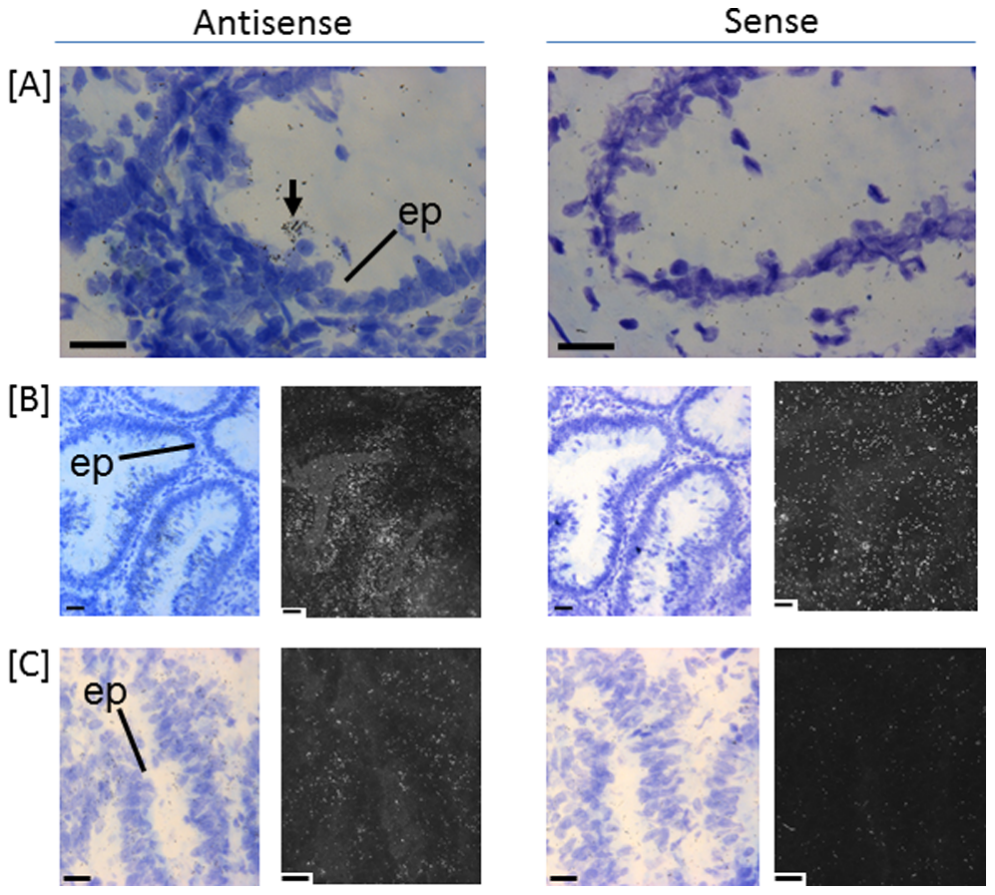
*In situ* hybridisation of *LGR5* transcripts in human colon (A) normal, (B) adenomatous polyp and (C) carcinoma. Emulsion autoradiographs showing expression of *LGR5* in discrete single cells (arrow) in epithelium (ep) in normal (A) and extensive expression in epithelium (ep) of adenomatous polyp (B) and carcinoma (C) in bright field and corresponding adjacent dark field images. Antisense hybridised tissue sections are shown to the left with sense hybridised tissue sections adjacent to the right (n = 5). Bar = 20 µm.

**Figure 5:**
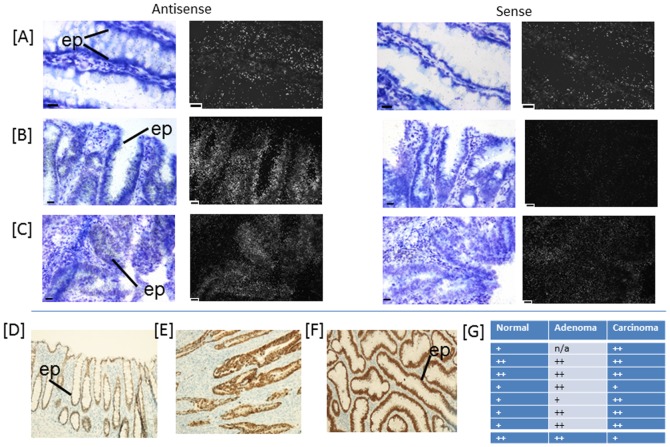
Localisation of *CDX2* transcripts and encoded protein in human colon. In situ hybridisation of CDX2 transcripts in human colon (A) normal, (B) adenomatous polyp and (C) carcinoma. Emulsion autoradiographs showing expression of *CDX2* in epithelium (ep) in bright field and corresponding adjacent dark field images in antisense (left) and sense (right) hybridised tissue sections (n = 5). Bar = 20 µm. (D) – (F) Representative paraffin-embedded tissue sections show immunohistochemical localisation of CDX2 expression in the human colon epithelium in (D) normal, (E) adenomatous polyp and (F) carcinoma. (G) Semi-quantitative scoring of staining intensity (increasing from + to +++) revealed increased immunostaining for CDX2 in adenomatous polyp and carcinoma (n = 8). Scoring system: + (detectable nuclear staining, weak), ++ (easily visible nuclear staining), n/a – no adenoma tissue in histological section.

**Figure 6:**
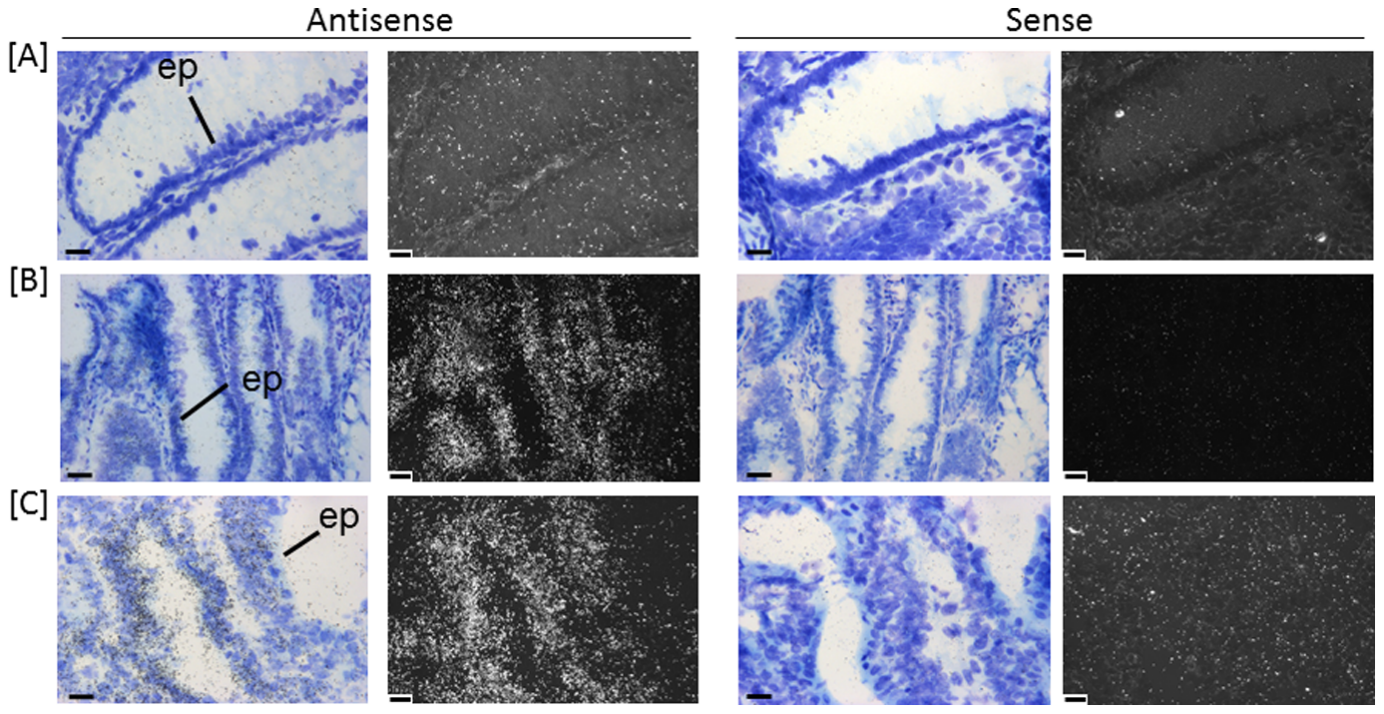
*In situ* hybridisation of *NOX1* transcripts in human colon (A) normal, (B) adenomatous polyp and (C) carcinoma. Emulsion autoradiographs showing expression of *NOX1* in epithelium (ep) in bright field and corresponding adjacent dark field images in antisense (left) and sense (right) hybridised tissue sections (n = 5). Bar = 20 µm.

**Figure 7:**
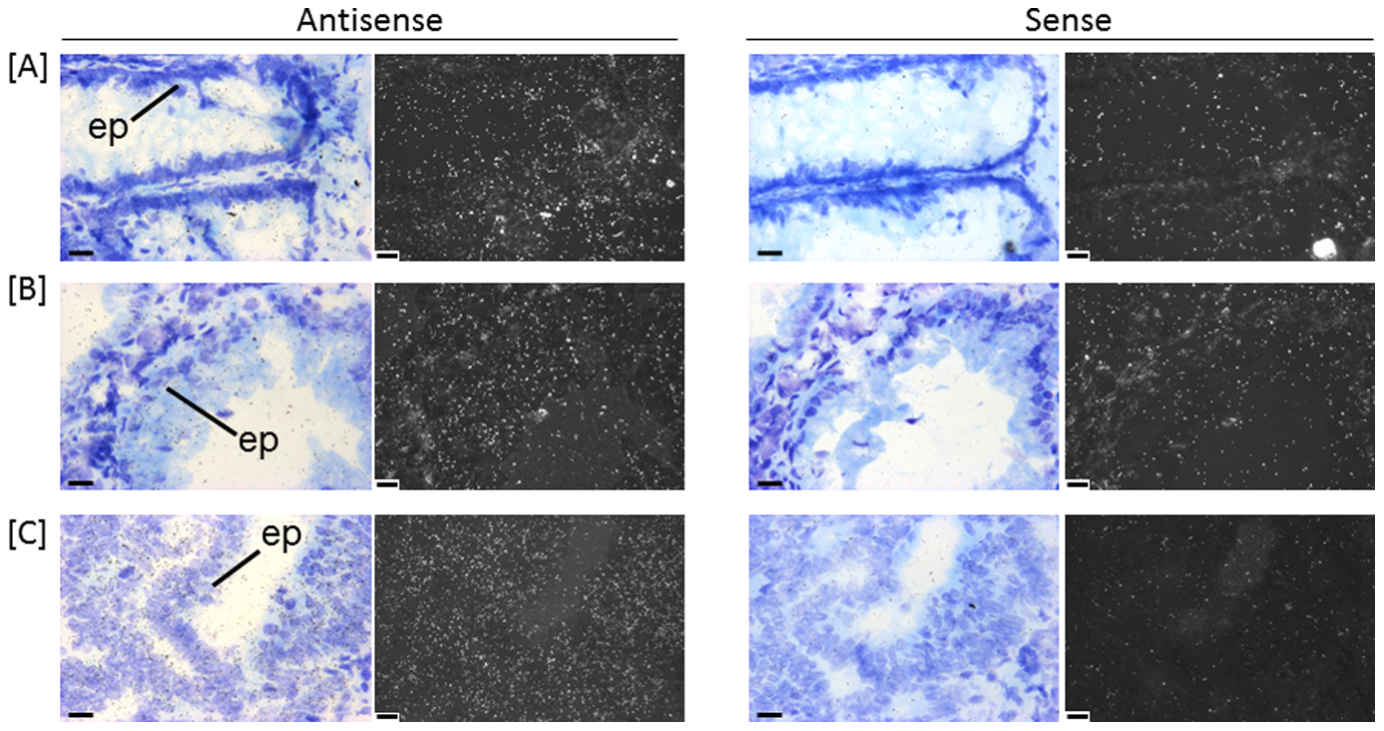
*In situ* hybridisation of *SLC9A2* transcripts in human colon (A) normal, (B) adenomatous polyp and (C) carcinoma. Emulsion autoradiographs showing expression of *SLC9A2* in epithelium (ep) in bright field and corresponding adjacent dark field images in antisense (left) and sense (right) hybridised tissue sections (n = 5). Bar = 20 µm.

Five “classifier genes”, *MS4A12*, *LGR5*, *CDX2*, *NOX1* and *SLC9A2*, contributing to distinguishing normal, adenoma and carcinoma tissue were further validated using *in situ* hybridisation to determine cellular localisation and distribution patterns of expression in normal, adenomatous polyp and carcinoma tissue (Figure 3–7). All five genes were expressed by epithelial cells in normal, adenomatous polyp and carcinoma. Expression levels in each tissue corroborated patterns indicated by the gene expression measured by hCellMarkerPlex. Notably, expression patterns were more diverse in carcinoma samples.

In normal tissues MS2A12 is expressed at high levels over the entire epithelia at the lumenal surface with reduced expression towards lower regions of the epithelium lining the colon crypts (Figure 3C). There is some expression apparent at the base of the crypts, but this is very much less than at the top. In contrast extensive epithelial expression in adenomatous polyps is absent and only a few crypts and lumenal epithelium in discrete localised areas exhibit MS4A12 expression (Figure 3D). Similarly, expression of MS4A12 is absent in most areas of carcinoma tissue, but when visible, in areas with more regular and differentiated epithelial structure, expression appears more intense than that seen over adenomatous polyp tissues (Figure 3E).

*In situ* hybridisation confirmed the low levels of *LGR5* expression in normal tissue, with localisation observed over discrete cells within the epithelium at the crypt base (Figure 4A). In constrast adenomatous polyps revealed extensive expression over the epithelium (Figure 4B). LGR5 expression was also expressed over areas of epithelium in carcinoma tissue, but expression levels were reduced in the carcinomas compared to adenomatous polyp (Figure 4C).

*CDX2* was expressed at low levels in epithelium lining the colon crypts in normal (Figure 5A) tissue and markedly increased in adenomatous polyp (Figure 5B) epithelium. Carcinoma tissue *CDX2* expression levels were either similar or less than those of adenomatous polyp epithelium (Figure 5C).

*NOX1* was expressed at low levels in epithelium lining the colon crypts in normal (Figure 6A) tissue, with higher levels in adenomatous polyp (Figure 6B) epithelium. Carcinoma tissue *NOX1* expression was variable with levels either similar to those of adenomatous polyp epithelium or markedly higher (Figure 6C).

*SLC9A2* expression was difficult to visualise and necessitated long exposure of *in situ* hybridised tissue sections (Figure 7). Similar patterns and level of expression were observed in normal (Figure 7A) and adenomatous polyp (Figure 7B) tissues with variable expression in carcinoma samples (Figure 7C).

### 3.6 CDX2 protein expression in colon normal, adenomatous polyp and carcinoma

Immunohistochemistry revealed CDX2 protein localisation (Figure 5D - 5F) that was consistent with *in situ* localisation of gene expression analysis (Figures 5A – 5C). CDX2 protein levels were generally similar or increased in epithelium of adenomatous polyps and carcinoma compared to normal colon tissue (Figure 5G). Staining ranged from weak increasing to easily visible nuclear staining (Figure 5G).

## Discussion

This study demonstrates a feasible strategy to develop objective classification of pathology status of colon biopsy tissue using bespoke assays to assess predictive gene signatures. Testing multiple markers at more than one location of adenomatous polyp and carcinoma clinical specimens is desirable to distinguish the variable dysplasia observed in clinical samples. GeXP assays facilitate this, requiring only nanogram quantities of total RNA to assess multiple selected gene targets, while remaining tissue can still be used for conventional pathological analysis in parallel. Total RNA extracted from archived surgical biopsy tissue samples is commonly of variable quality. However, as observed previously [12] and in the present study, it is possible to obtain GeXP assay gene expression profiles from total RNA extractions that have low RIN values. The ability to measure multiple markers simultaneously within one total RNA sample generates more comprehensive data to assess pathological status, particularly if the data for a one marker is compromised as a consequence of sample quality, inter-individual or within biopsy variation.

Gene expression profiling by hCellMarkerPlex assay identified potential classifier genes that contributed markedly to classification of tissue pathology status (Figure 1). Notably all five classifier genes selected for further analysis, *MS4A12*, *LGR5*, *CDX2*, *NOX1* and *SLC9A2*, are expressed by epithelial cells (Figures 3–7). The aberrant expression of these gene markers is associated with the observed profound alteration in the microarchitecture of the colon epithelium, the origin of adenomatous polyp formation and most carcinomas in the colon (Figures 3–7).

MS4A12 is a cell surface protein found to be predominantly expressed at the apical surface of the colon epithelium [22]. This is supported by the localisation of *MS4A12* gene expression reported in this study (Figure 3D). It has been proposed that MS4A12 inhibits cell proliferation and motility associated with differentiation by regulating store operated calcium channels [22], [23]. Expression has been reported to be specific for colon normal and carcinoma tissues with no detectable expression in breast, lung, prostate, gastric, renal, malignant melanoma, hepatocellular, leukemia and head neck cancer[22]. Koslowski *et al*. [22] report variable expression of the MS4A12 protein in carcinoma samples supported by the variable localisation of gene expression in this study. It is significant to note in this study the first report of the highly significant reduction in *MS4A12* gene expression in adenomatous polyps. This potentially reflects the greater heterogeneity of tissue sampled from extensive carcinomas that may contain samples with varying degrees of dysplasia. Regions within a carcinoma may exhibit a near normal morphology and patterns of gene expression that have a greater degree of similarity with normal tissues.

*MS4A12* is reported to be regulated by a CDX2 responsive promotor [23]. CDX2 is known to regulate gut specific genes and processes determining differentiation of gut epithelium [24]. Notably, *CDX2* was identified in this study as a potential classifier gene with significantly elevated mean expression levels in adenomatous polyp and carcinoma compared to normal tissue. However, while most adenomatous polyps tested revealed elevated expression of *CDX2* compared to matched normal tissue, the carcinoma samples were more variable with some carcinoma having comparable levels with normal tissue that are consequently reduced compared to adenomatous polyp. Notably immunohistochemistry also established elevated expression of CDX2 protein in adenomatous polyps (Figure 6E, 6G). Hence, despite up-regulation of CDX2 gene and protein expression, *MS4A12* transcription, regulated by CDX2, is down-regulated in adenomatous polyp. More detailed gene expression analysis revealed that both transcript variants of *MS4A12* are regulated in a similar pattern within tissue samples. Hence, down-regulation of *MS4A12* is attributed to loss of transcription of both variants equally (Figure 3A, 3B). The results of this study support the complex relationship between CDX2 and colon carcinogenesis reported in previous studies [23], [25], [26]. Loss of *CDX2* regulated gene transcription is likely to be an important factor in the transition from ordered epithelium in normal tissue as opposed to that of adenomatous polyps.

*LGR5* is a colon stem cell marker [27] and its up-regulation is implicated in uncontrolled proliferation of the epithelium in colon carcinogenesis associated with β-catenin signalling [28]. It is considered to be a potential marker for colon cancer stem cells and is linked to progression and poor prognosis in colon cancer patients [29]. In the present study mean expression levels are significantly higher in adenomatous polyp samples compared to either normal or carcinoma. *LGR5* expression is sparse and limited to discrete single cells in the epithelial layer near the base of colon crypts in normal tissue (Figure 4A). It is clear that the number of *LGR5* expressing cells are markedly increased and easily detected in the epithelium of adenomatous polyps and carcinoma. *In situ* localisation reported here supports the contention that *LGR5* is intimately linked to either increased proliferation of the colon stem cells or a failure to down-regulate LGR5 and initiate differentiation (Figure 4). The significance of the higher levels of *LGR5* in epithelium of adenomatous polyps requires further investigation to determine potential links with progression of carcinogenesis.

*NOX1* is a member of the NADPH oxidase family of enzymes and generates superoxide and H_2_O_2_. Production of the reactive oxygen species produced can generate second messengers to suppress apoptosis [30]. Over expression of *NOX1* can result in excessive generation of reactive oxygen species linked to cancer. Activation of angiogenesis has been associated with over expression of *NOX1* in aggressive carcinomas [31].

Evidence obtained from this study indicates profound changes in epithelial gene transcription programming during development of adenomatous polyps that have similarities with carcinomas. Indeed aberrant gene transcription of some markers associated with carcinogenesis, *NOX1*, *LGR5*, *MS4A12*, [28], [31], [32] reveal greatest deviation from that of normal tissue in the adenomatous polyps prior to development of carcinoma. Other markers, *EZR* and *KRT18* [32], [33] already exhibit characteristics of the aberrant expression observed in carcinoma within the adenomatous polyps. It may be inferred that transcriptional changes in adenomatous polyps predispose them to carcinogenesis. This is characterised by increased *NOX1* and *LGR5* expressing cells that indicate a proliferative phenotype with concomitant loss of *MS4A12* and *EZR* indicating inhibition of differentiation and loss of epithelial cell structure. Interestingly, high *COL1A1* expression, a fibroblast marker, appears to contribute to differentiate carcinomas with significantly lower expression of this marker in adenomatous polyps and normal tissues.

Consequently, the identified gene signatures can provide objective classification of diseased tissue and provide some insight on altered programming of epithelial gene transcription that precedes or delineates carcinoma development. This study has established the concept of using classifier genes to develop gene signature assays to provide objective classification of health and disease status of colon biopsy specimens. This will facilitate further design and development of multiplex assays that can assist pathologists to make objective decisions on disease initiation, staging, progression and responses to treatment.

## Supporting Information

Table S1 Patient tissue samples.
(DOC)

Table S2 Genes and primers used in the GeXP hCellMarkerPlex RT-PCR. Reference genes (bold) are used for normalisation and calculation of relative gene expression levels. A synthetic internal reverse transcription and PCR amplification control target is also incorporated (*italic*).
(DOC)
