# Supplementary material for: Predictive Gene Signatures: Molecular Markers Distinguishing Colon Adenomatous Polyp and Carcinoma
Source: PLoS One. 2014 Nov 25;9(11):e113071. doi: 10.1371/journal.pone.0113071 (PMC4244109; doi:10.1371/journal.pone.0113071)
Supplement: Table S1 — Patient tissue samples. (DOC) [file pone.0113071.s001.doc]

Suppl figure Table S1 Patient tissue samples

| **Sample type** | **Age at Surgery** | **Sex** | **Ethnic Group** | **Reason for Surgery** | **Location of carcinoma** | **Location of adenoma** | **Dukes Classification** | **TNM staging** | **Adenoma subtype** | **Adenoma Size** | **RIN** |
| --- | --- | --- | --- | --- | --- | --- | --- | --- | --- | --- | --- |
| Normal | 55 years 3 months | M | White | Biopsy proven cancer | Caecum |  | B | T4 N0 Mx |  |  | 5 |
| Carcinoma |  |  |  |  |  |  |  |  |  |  | 9.7 |
| Normal | 62 years 8 months | M | White | Biopsy proven cancer | Descending colon |  | C | T3 N2 Mx |  |  | 8.3 |
| Carcinoma |  |  |  |  |  |  |  |  |  |  | 8.6 |
| Normal | 81 years 6 months | M | White | Biopsy proven cancer | Ascending colon |  | B | T3 N0 Mx |  |  | 5.1 |
| Carcinoma |  |  |  |  |  |  |  |  |  |  | 9.6 |
| Normal | 63 years 3 months | M | White | CT scan indicated cancer | Caecum |  | C1 | T3 N2 Mx |  |  | 9.6 |
| Carcinoma |  |  |  |  |  |  |  |  |  |  | 10 |
| Normal | 74 years 11 months | F | White | CT scan indicated cancer | Ileum |  | C1 | T4 N2 Mx |  |  | 7.8 |
| Carcinoma |  |  |  |  |  |  |  |  |  |  | 9.7 |
| Normal | 73 years 4 months | M | White | Biopsy suggestive of cancer | Ascending colon |  | C1 | T1 N1 Mx |  |  | 2.2 |
| Carcinoma |  |  |  |  |  |  |  |  |  |  | 5.3 |
| Normal | 37 years 11 months | M | White | Biopsy proven cancer | Sigmoid |  | B | T3 N0 Mx |  |  | 5.8 |
| Carcinoma |  |  |  |  |  |  |  |  |  |  | 2.3 |
| Normal | 74 years 6 months | F | White | Biopsy proven cancer | Caecum |  | B | T3 N0 Mx |  |  | 5 |
| Carcinoma |  |  |  |  |  |  |  |  |  |  | 8.5 |
| Normal | 75 years 2 months | F | White | Biopsy proven cancer | Sigmoid |  | B | T3 N0 Mx |  |  | 6.5 |
| Carcinoma |  |  |  |  |  |  |  |  |  |  | 9.3 |
| Normal | 76 years 4 months | F | White | Biopsy proven cancer | Caecum |  | B | T3 N0 Mx |  |  | 8.2 |
| Carcinoma |  |  |  |  |  |  |  |  |  |  | 9.9 |
| Normal | 76 years 3 months | F | White | Biopsy proven adenoma |  | Sigmoid |  |  | Tubulovillous adenoma | 25mm | 8.2 |
| Adenoma |  |  |  |  |  |  |  |  |  |  | 9.1 |
| Normal | 72 years 5 months | F | White | Biopsy proven adenoma |  | Caecum |  |  | Adenoma | 39mm | 10 |
| Adenoma |  |  |  |  |  |  |  |  |  |  | 9.4 |
| Normal | 77 years 10 months | F | White | Biopsy proven adenoma |  | Caecum |  |  | Tubulovillous adenoma | 107 x 76mm | 6.8 |
| Adenoma |  |  |  |  |  |  |  |  |  |  | 9.6 |
| Normal | 76 years 3 months | M | White | Biopsy proven adenoma |  | Sigmoid |  |  | Tubulovillous adenoma | 82mm | 5.7 |
| Adenoma |  |  |  |  |  |  |  |  |  |  | 9.5 |
| Normal | 60 years 4 months | M | White | Biopsy proven adenoma |  | Rectum |  |  | Tubulovillous adenoma | 26mm | 9.6 |
| Adenoma |  |  |  |  |  |  |  |  |  |  | 9.6 |
| Normal | 54 years 6 months | M | White | Biopsy proven adenoma |  | Caecum |  |  | Tubulovillous adenoma | 32 x 14 x 12mm | 9.4 |
| Adenoma |  |  |  |  |  |  |  |  |  |  | 9.4 |
| Normal | 60 years 10 months | F | White | Biopsy proven cancer + adenoma | Ascending colon | Caecum | C1 | T3 N1 Mx | Adenoma | 22mm | 4 |
| Adenoma |  |  |  |  |  |  |  |  |  |  | 8.7 |
| Carcinoma |  |  |  |  |  |  |  |  |  |  | 9.2 |
| Normal | 58 years 8 months | M | White | Biopsy proven cancer | Sigmoid | Sigmoid | C1 | T3 N1 Mx | Adenoma | Not known | 6.5 |
| Polyp |  |  |  |  |  |  |  |  |  |  | 9.5 |
| Carcinoma |  |  |  |  |  |  |  |  |  |  | 7.1 |
| Normal | 79 years 7 months | M | White | Biopsy proven cancer + adenoma | Transverse colon | Rectum | B | T3 N0 M0 | Tubular adenoma | 34 x 20 x 6mm | 7.3 |
| Polyp |  |  |  |  |  |  |  |  |  |  | 8.5 |
| Carcinoma |  |  |  |  |  |  |  |  |  |  | 9.6 |
| Normal | 77 years 7 months | M | White | Biopsy proven cancer | Hepatic flexure | Caecum | B | T3 N0 Mx | Adenoma | 45 x 14 x 40mm | 9.1 |
| Polyp |  |  |  |  |  |  |  |  |  |  | 9.7 |
| Carcinoma |  |  |  |  |  |  |  |  |  |  | 6.5 |
| Normal | 76 years 4 months | M | White | Not known - presumed cancer diagnosis | Transverse colon | Hepatic flexure | B | T3 N0 Mx | Adenoma | 10mm | 3.8 |
| Polyp |  |  |  |  |  |  |  |  |  |  | 7.5 |
| Carcinoma |  |  |  |  |  |  |  |  |  |  | 7.4 |
| Normal | 65 years 11 months | M | White | Not known - presumed cancer diagnosis | Sigmoid colon | Sigmoid | A | T2 N0 Mx | Adenoma | Not known | 7.9 |
| Polyp |  |  |  |  |  |  |  |  |  |  | 9.2 |
| Carcinoma |  |  |  |  |  |  |  |  |  |  | 2 |
| Normal | 59 years 3 months | M | White | Biopsy proven cancer + polyp | Sigmoid colon | Sigmoid | B | T3 N0 Mx | Tubulovillous adenoma | 16mm | 8 |
| Polyp |  |  |  |  |  |  |  |  |  |  | 8.7 |
| Carcinoma |  |  |  |  |  |  |  |  |  |  | 4.3 |
| Normal | 70 years 11 months | M | White | Biopsy proven cancer + polyp | Rectum | Recto-sigmoid | B | T3 N0 Mx | Adenoma | 8mm | 8.3 |
| Polyp |  |  |  |  |  |  |  |  |  |  | 9 |
| Carcinoma |  |  |  |  |  |  |  |  |  |  | 8.6 |
| Normal | 55 years 11 months | F | White | Not known - presumed cancer diagnosis | Ascending colon | Ascending colon | B | T3 N0 Mx | Sessile serrated polyp | 12mm | 4.6 |
| Polyp |  |  |  |  |  |  |  |  |  |  | 4.7 |
| Carcinoma |  |  |  |  |  |  |  |  |  |  | 9 |
| Normal | 50 years 5 months | M | White | Not known - presumed cancer diagnosis | Ascending colon | Not stated | B | T3 N0 | Adenoma | Not known | 7 |
| Polyp |  |  |  |  |  |  |  |  |  |  | 7.7 |
| Carcinoma |  |  |  |  |  |  |  |  |  |  | 8.7 |
| Normal | 75 years 3 months | F | White | CT scan suggested malignancy | Splenic flexure | Sigmoid | B | T3 N0 Mx | Adenoma | 34mm | 6.3 |
| Polyp |  |  |  |  |  |  |  |  |  |  | 9.3 |
| Carcinoma |  |  |  |  |  |  |  |  |  |  | 9.1 |
| Normal | 74 years 9 months | M | White | Biopsy proven cancer | Ascending colon | Caecum | B | T3 N0 Mx | Moderately dysplastic tubulovillous adenoma | 15mm | 4.8 |
| Polyp |  |  |  |  |  |  |  |  |  |  | 4.6 |
| Carcinoma |  |  |  |  |  |  |  |  |  |  | 9 |
| Normal | 69 years 2 months | F | White | CT scan suggested malignancy | Caecum | Rectum | B | T3 N0 Mx | Serrated adenoma | 12mm | 3 |
| Polyp |  |  |  |  |  |  |  |  |  |  | 4.5 |
| Carcinoma |  |  |  |  |  |  |  |  |  |  | 4.8 |
| Normal | 82 years 1 month | M | White | Biopsy proven cancer | Recto-sigmoid | Lower rectum | A | T1 N0 Mx | Low grade dysplastic tubulovillous adenoma | 51mm | 8.4 |
| Polyp |  |  |  |  |  |  |  |  |  |  | 9.5 |
| Carcinoma |  |  |  |  |  |  |  |  |  |  | 4.3 |
