# Supplementary material for: Predictive Gene Signatures: Molecular Markers Distinguishing Colon Adenomatous Polyp and Carcinoma
Source: PLoS One. 2014 Nov 25;9(11):e113071. doi: 10.1371/journal.pone.0113071 (PMC4244109; doi:10.1371/journal.pone.0113071)
Supplement: Table S2 — Genes and primers used in the GeXP hCellMarkerPlex RT-PCR. Reference genes (bold) are used for normalisation and calculation of relative gene expression levels. A synthetic internal reverse transcription and PCR amplification control target is also incorporated (italic). (DOC) [file pone.0113071.s002.doc]

Suppl figure Table S2: Genes and primers used in the GeXP hCellMarkerPlex RT-PCR. Reference genes (bold) are used for normalisation and calculation of relative gene expression levels. A synthetic internal reverse transcription and PCR amplification control target is also incorporated (*italic*).

| Accession Number | Gene Symbol |  | Left Primer Sequence | Right Primer Sequence | Product size with Universals | | Reverse Primer Dilution (nM) |
| --- | --- | --- | --- | --- | --- | --- | --- |
| NM_001615 | ACTG2 |  | TGGATCAGCAAGCCTGAGTA | TAGTCTCGAGGGGATCCTTG | | 137 | 62.5 |
| NM_000552 | VWF |  | CAGTACGTTCTGGTGCAGGA | ACCCGTTTCTTGCATTTCAC | | 144 | 500 |
| NM_001111077 | EZR |  | ATACACTGCCAAGATTGCCC | TCCTTGGTCTTCACCAGGTC | | 151 | 62.5 |
| NM_013955 | NOX1 |  | TTAACAGCACGCTGATCCTG | TGTGGAAGGTGAGGTTGTGA | | 157 | 500 |
| NM_004964 | HDAC1 |  | TGGAAATCTATCGCCCTCAC | TCTCTGCATCTGCTTGCTGT | | 165 | 500 |
| **NM_181838** | **UBE2D2** |  | **CAGCACAGTGTTCAGCAGGT** | **TGAAGGGGTAATCTGTTGGG** | | **172** | **15.6** |
| NM_053056 | CCND1 |  | GCTGTGCATCTACACCGACA | CTCTGGCATTTTGGAGAGGA | | 179 | 500 |
| NM_153446 | B4GALNT2 |  | CCTTCAAGCAGTGTTCAGCA | TTGGCTTCACATTTGCACTG | | 193 | 62.5 |
| NM_001299 | CNN1 |  | GAGGAGGGAAGAGTGTGCAG | TACTTCTGGGCCAGCTTGTT | | 207 | 1000 |
| NM_003047 | SLC9A2 |  | TGCTCCAGAACCTGCTCTTT | CTGGCAAAACGACTTGAACA | | 213 | 500 |
| NM_001927 | DES |  | CATCGCGGCTAAGAACATTT | GGAATCGTTAGTGCCCTTCA | | 221 | 1000 |
| NM_003667 | LGR5 |  | AGAATTTGCGAAGCCTTCAA | TATTTTGTTCAGGGCCAAGG | | 228 | 1000 |
| NM_000088 | COL1A1 |  | CCACTTGCTTGAAGACCCAT | GGTGTTTGAGCATTGCCTTT | | 235 | 500 |
| NM_182649 | PCNA |  | TGTAAACCTGCAGAGCATGG | TACTAGCGCCAAGGTATCCG | | 242 | 500 |
| NM_001804 | CDX1 |  | TCGGACCAAGGACAAGTACC | CTGTTGCTGCTGCTGTTTCT | | 248 | 500 |
| NM_017716 | MS4A12 |  | CAACAGCCTCTGGGTTCAAT | AATGTGCATCAATCCAACCA | | 256 | 500 |
| NM_199187 | KRT18 |  | TAGATGCCCCCAAATCTCAG | GAGTCCAGGTCGATCTCCAA | | 263 | 500 |
| NM_002961 | FSP1 |  | TCTCTCCTCAGCGCTTCTTC | GCTGTCCAAGTTGCTCATCA | | 276 | 1000 |
| NM_002457 | MUC2 |  | TCAAGTCCGATGGCAGTGTA | CCGTCTTGAAGTCGTCACCT | | 283 | 1000 |
| **NM_004048** | **B2M** |  | **AGGCTATCCAGCGTACTCCA** | **GTTCACACGGCAGGCATACT** | | **291** | **15.6** |
| NM_004822 | NTN1 |  | AAAACCTGCAACCAAACCAC | GTGGATCTGGACGGCATAGT | | 298 | 1000 |
| NM_001265 | CDX2 |  | GAACCTGTGCGAGTGGATG | TTGCTGCTGCAACTTCTTCT | | 305 | 1000 |
| NM_032991 | CASP3 |  | TGGAATATCCCTGGACAACAG | GGCTCAGAAGCACACAAACA | | 312 | 1000 |
|  | *Kan(r)* |  | ATCATCAGCATTGCATTCGATTCCTGTTTG | ATTCCGACTCGTCCAACATC | | 325 | 500 |
